# 

**Published:** 1921-06-25

**Authors:** 


June 25, 1921] [Price Twopence

### The Hospital

The Workers' Newspaper of Administrative Medicine and Institutional Life, Administration, National Insurance and Health.

CONTENTS

**The Census** ... ... ... ... ... ... 207

**Post-Graduate Medical Education...** ... ... 207

**Hospital Facilities for the Well-to-Do** ... ... 208
The Private Clinic System in Great Britain.

**Notes of the Week** ... ... ... ... .. 209
Ireland and Her Hospitals—Mr. Walter Alvey's Successor—A Jubilee at 800 Years—Hospital Managers in Conference—The Reception of the Cave Report—A Hospital Pioneer—A Record Gift and a New Policy—A Hospital Chairman's Bequests—Co-ordination in Gloucester.

**Deficiency Diseases** ... ... ... ... ... 211

A New Light on Vitamines.

**The Nile from the Point of View of Health** ... 212

**Celluloid Splints** ... ... ... ... ... 213

Their Uses and Manufacture.

**The Public Well-Being** ... ... ... ... ... 215

Medical Secrecy—Dentistry and the Dentists Bill—Games for Girls—The International Journal of Public Health.

**Incipient Insanity and Home Life** ... ... ... 214

**The Institutional Library** .. ... ... ... 217
Diseases of the Throat, Nose, and Ear—Transactions of the National Association for the Prevention of Tuberculosis—Injuries of the Peripheral Nerves—Some Conclusions on Cancer.

**The Editor's Letter-Box** ... ... ... ... 218
Prevention of V.D.—Registration of Fever Nurses.

**The Matrons' and Sisters' Department** ... ... 219
Scotland and the College.
A University Diploma for Nurses.

**Round the Hospitals** ... ... ... ... 220

**Nursing Diploma of the University of Leeds** ... 222

**Welfare Work in Jeopardy** ... ... ... ... 222

**Passing Events and Topics** ... ... ... ... 223

**Gifts and Bequests** ... ... ... ... ... 223

**The College of Nursing (Limited by Guarantee)** 224

**Matrons' and Sisters' Appointments** ... ... 224

